# Addressing the policy cacophony does not require more evidence: an argument for reframing obesity as caloric overconsumption

**DOI:** 10.1186/1471-2458-12-1042

**Published:** 2012-11-30

**Authors:** Jacob J Shelley

**Affiliations:** 1 Doctor of Juridical Science Candidate, Faculty of Law, University of Toronto, 84 Queen’s Park, Toronto, ON M5S 2C5, Canada

**Keywords:** Obesity, Caloric overconsumption, Framing, Food environment, Public health policy

## Abstract

**Background:**

Numerous policies have been proposed to address the public health problem of obesity, resulting in a policy cacophony. The noise of so many policy options renders it difficult for policymakers to determine which policies warrant implementation. This has resulted in calls for more and better evidence to support obesity policy. However, it is not clear that evidence is the solution. This paper argues that to address the policy cacophony it is necessary to rethink the problem of obesity, and more specifically, how the problem of obesity is framed. This paper argues that the frame “obesity” be replaced by the frame “caloric overconsumption”, concluding that the frame caloric overconsumption can overcome the obesity policy cacophony.

**Discussion:**

Frames are important because they influence public policy. Understood as packages that define issues, frames influence how best to approach a problem. Consequently, debates over public policy are considered battles over framing, with small shifts in how an issue is framed resulting in significant changes to the policy environment. This paper presents a rationale for reframing the problem of obesity as caloric overconsumption. The frame “obesity” contributes to the policy cacophony by including policies aimed at both energy output and energy input. However, research increasingly demonstrates that energy input is the primary cause of obesity, and that increases in energy input are largely attributable to the food environment. By focusing on policies that aim to prevent increases in energy input, the frame caloric overconsumption will reduce the noise of the obesity policy cacophony. While the proposed frame will face some challenges, particularly industry opposition, policies aimed at preventing caloric overconsumption have a clearer focus, and can be more politically palatable if caloric overconsumption is seen as an involuntary risk resulting from the food environment.

**Summary:**

The paper concludes that policymakers will be able to make better sense of the obesity policy cacophony if the problem of obesity is reframed as caloric overconsumption. By focusing on a specific cause of obesity, energy input, the frame caloric overconsumption allows policymakers to focus on the most promising obesity prevention policies.

## Background

Much ink has been spilled trying to justify policies and interventions to prevent obesity. Although obesity has been a public health issue for some time, public awareness and political attention has grown exponentially in the last decade as the health, social, and economic consequences of obesity became more apparent [1-3]. Despite this wide recognition [1,4,5], ample resistance has nevertheless been mounted against obesity prevention by the food industry [6], those opposed to the ‘anti-fat’ movement [7,8], or those who simply wish to prevent the ever-expanding role of the state in private affairs [9-12]. Such opposition is not new to public health. There has long been resistance to public health interventions, which often are decried as paternalistic and contrary to civil liberties [13,14]. But even if unanimous support existed for implementing policies and interventions to combat obesity – which remains an unlikely accomplishment – there would remain a vociferous debate about what types of measures would be most effective and thus worth implementing. In light of this debate, respected health organizations are calling for more evidence to support obesity policy. For example, the 2010 report by the Institute of Medicine, entitled *Bridging the Evidence Gap in Obesity Prevention*, explicitly addresses the need to adopt the L.E.A.D. framework (**L**ocate Evidence, **E**valuate Evidence, **A**ssemble Evidence, and Inform **D**ecisions) to ensure evidence-based decision-making in obesity prevention and offers suggestions on how to better generate and evaluate evidence [15]. However, it is not clear that a lack of evidence is the underlying problem inhibiting the implementation of obesity prevention policies, or the solution to garnering support for such policies.

In fact, the generation of more evidence may simply exacerbate what Lang and Rayner have termed a policy cacophony on obesity [16]. A policy cacophony exists when different policy solutions have been developed to address a single issue, and then must compete with one another for support, funding, and implementation [16]. With competing theories about the root causes of obesity, and many plausible although limited solutions being proffered, the resulting “noise” renders it nearly impossible to determine what policies would be most effective. Consequently, as policymakers call for stronger evidence, the evidence continues to support competing policy options, leaving policymakers unsure of which policies to implement. More evidence may simply exacerbate the obesity policy cacophony, especially given the difficulty of translating evidence into policy. Indeed, Lang and Rayner suggest that while the scientific understanding of obesity has become more sophisticated, the policy cacophony has become more muddled [16]. They argue that policymakers must move away from making small steps or implementing single solutions, and instead adopt a “big thinking, many changes” approach, suggesting a need to reconceptualize the basis on how to tackle obesity [16]. It is not clear, however, that “big thinking” about obesity policy will be sufficient, as it is likely to simply inject more policy options – albeit more complex and possibly more promising policy options – into an already overcrowded market place of ideas.

Rather than big thinking or simply generating more evidence, both of which will only further muddle the policy cacophony, this paper argues that we need to rethink how we approach the problem of obesity. More specifically, there is a need to rethink how the problem of obesity is framed. In the past few years, increased attention has been paid to the framing of obesity. Primarily, research has focused on the framing of obesity in the media [3,17-20], although studies have also examined the framing of obesity by the food and beverage industry [21], key interest groups [1,3], and policy makers [22]. The central argument of this paper is that how the problem of obesity is currently framed contributes to and exacerbates the difficulty of interpreting evidence and sifting through the noise of policy cacophony. The problematic frame is “obesity”, and this paper contends that much could be gained by adopting the frame “caloric overconsumption.” As a frame, caloric overconsumption provides clearer answers to core questions, such as who is responsible for obesity [2], which will help shape the overall policy approach [18], and will allow researchers and policymakers to make better use of proposed frameworks, such as L.E.A.D. [15]. The proposed frame also draws attention to the role of toxic food environments, and the involuntary risk of overconsumption they promote.

The paper begins with a discussion of the importance of frames. It then examines the rationale for reframing, first identifying the problems with the current frame obesity before exploring the benefits of the proposed frame caloric overconsumption. Next, it considers the promise of the frame caloric overconsumption, as well as some of the challenges that reframing will present. The paper concludes that reframing obesity as caloric overconsumption is the type of change that is needed to address the obesity policy cacophony.

## Discussion

### The importance of framing

The importance of framing is widely recognized, especially given the potential for frames to influence the policy environment [22]. Frames have been described as a package that promotes a particular definition of an issue [22]. More than defining problems, they also diagnose the causes of the problems, make moral judgments, and suggest resolutions [23]. Frames are also used to identify who is affected by a particular problem as well as who bears responsibility for resolving a problem [19,22]. If frames shape the inferences individuals make about a message [24], “framing” is the process that influences how individuals develop a particular conceptualization or reorient their thinking about a particular issue [25]. The public is therefore subject to framing competitions waged by stakeholders who wish to control public opinion about an issue. Multiple actors contribute competing frames, because frames will dictate what response is required. Consequently, debates over public policy have been characterized as battles for framing [26]. These framing competitions can have negative implications for forming policy, as advocates can become more concerned with promoting a preferred conceptions of the problem than with identifying the most appropriate and effective response [27].

Once a frame is accepted it will influence how to best approach the problem. Stakeholders thus have a vested interest in promoting a particular frame as superior [21]. Strong frames emerge when they are perceived as the more compelling argument, or when they are thought to more accurately correspond with reality [21,25]. In situations where empirical reality is complex, as is the case with obesity, strong frames often emerge due to a claimant’s rhetorical skill and credibility, and not because of evidence [3]. Chong and Druckman argue that this can be a problem, as strong frames might be built on exaggerations or even outright lies that play on public fears and prejudices [25]. In framing competitions considerable effort is expended to control the frame because of a phenomenon known as “framing effects.” Framing effects occur when small changes in how an issue is presented result in large changes of opinion [25]. Importantly, alternative ways of phrasing an issue, even if modest, can significantly alter how an issue is understood. Research indicates that *reframing* an issue can shift public opinion, as well as the policymaking environment [19]. In public health, tobacco control is often used as an example of how significant policy shifts can result from small changes in how an issue is framed [19,28], although the importance of framing has been recognized in a wide spectrum of public health issues, from HIV testing [29] to gambling [30].

### Reframing obesity

In order to make sense of the obesity policy cacophony, this paper argues that the problem of obesity should be reframed as caloric overconsumption. There are two broad rationales for this reframing. The first rationale deals with the problems accompanying the current frame obesity. In addition to having become politicized, obesity is an outcome and not a cause^a^. As a frame, “obesity” does not identify any specific causes – and obesity certainly is not the cause of itself! Thus the frame obesity remains open to be interpreted and influenced by competing theories about what does cause obesity. This makes it difficult to identify or assess potential policies or interventions. The second rationale stems from the potential benefits of using the proposed frame, caloric overconsumption. The frame caloric overconsumption minimizes some of the framing competition by identifying a specific cause of obesity, energy input. Moreover, the frame caloric overconsumption will permit a more critical analysis of the various policies and interventions that can be used in obesity prevention.

Part of the problem with the frame obesity is that it is highly politicized [31]. While politicization has increased attention to the problem of obesity, it has also spurred on the vociferous debate. There has been an ongoing discussion about the appropriateness of calling obesity an “epidemic”^b^[31-34], with critics arguing “epidemic” is simply used to capture the attention of media and policy makers [9,11,27,33]. There has been controversy over the use of the population measure of obesity, body-mass index, which critics suggest is too crude of a tool to be useful [35], and can be used to bolster claims about the epidemic nature of obesity [11]. Some have even suggested obesity is a myth perpetuated the weight-loss industry [32], including advocacy groups representing the restaurant and fast-food industry [21,36]. Controversy resulting from the politicization of obesity has led some to question whether or not obesity is even a problem that needs to be addressed [33], shifting focus away from what types of interventions or policies might be worth implementing.

A more significant problem with the frame obesity is obesity’s complex etiology. As Mann has observed, the reality of obesity is too complex to offer an integrated explanation [37]. Different disciplines offer a particular lens into the problem, but each is limited, confined to the epistemological and scientific underpinnings of the discipline. Given this complexity, with numerous causal pathways, it is inevitable that solutions to address obesity will also be complex [38]. Add to the complex causes of obesity the frames proffered by industry, advocacy groups, and the media, and the resulting policy cacophony is not surprising. As Schlesinger notes, “it is easy to imagine policy makers becoming so enmeshed in complex contests about the meaning of obesity that they can never move on to designing appropriate remedies” [27](p. 787).

The framing contest is perhaps most obvious when considering the commonly noted formula that obesity is the result of a sustained caloric imbalance, where energy input exceeds energy output [39]. While empirically accurate, and seductively simple [40], this formula is a great oversimplification and prone to distortion and erroneous assumptions [41]. Consider, for example, how the food and beverage industry has focused on the role of physical activity (energy output) [1,6,42], often making little to no mention of the environmental determinants of obesity [1]. There is even an outright rejection by some that overeating or energy input contributes to obesity [21,36]. At most, industry groups emphasize energy balance, where the role of physical activity is on par with energy consumption [43]. This corresponds with the industry’s position that there are no good foods or bad foods^c^, provided individuals expend an amount of energy equivalent to or greater than the amount of energy they consume. To the extent that industry does recognize energy input’s contribution to obesity, the focus is on personal and parental responsibility^d^, both in consumption behaviours and physical activity [1]. Unsurprisingly, the industry has been found to support frames that promote autonomy, individual choice, and “common sense” consumption [21].

However, research is increasingly demonstrating that energy output actually plays a very small role in obesity [44-49]. Recent estimates suggest that 60% to 100% of obesity amongst Canadians is related to excess calorie consumption, and not inadequate energy expenditure [44]. In fact, exercise and physical activity has been shown to play a limited – and sometimes counterproductive – role in weight control [50]. While some research has shown that energy output can result in equal or greater weight loss than might be achieved with an equivalent reduction in energy input [41], increased physical activity can be accompanied by increases in energy intake in free living adults [51]. This is not to suggest that exercise or physical activity is not important. It certainly is, and for a variety of reasons, including physical and mental health. But as a strategy for obesity prevention (or treatment), the evidence does not support physical activity as a promising solution, particularly at a population level. The proposed frame, caloric overconsumption, has the potential to change the policy environment by focusing on a specific cause of obesity, energy input.

If obesity can largely be attributed to caloric overconsumption, the discussion about obesity prevention policies should focus on the reasons why energy input has increased. One way to evaluate increases in energy input is to assess dietary behaviours. Diet is widely perceived to be a matter of personal responsibility, although there is little evidence to support this position [1,37,52,53]. A review of the literature examining psychosocial determinants of dietary behaviour found generally low predictiveness (R^2^<0.3) [54], reflecting the fact that there are many factors that influence dietary behaviours, such as local food availability, food affordability, and social and economic environments [55,56]. Indeed, in Canada, only 0.5% of Canadians were found to have an adequate diet using a comprehensive diet quality indicator [57]. It seems unlikely that 99.5% of Canadians are consciously choosing to have less than optimal diets. The more obvious answer for why there has been an increase in energy input is that it is the consequence of the food environment [46].

Consider the fact that in 2002, 530 more calories were available for consumption in Canadians’ diets than in 1985 [44,58], an alarming amount considering that an excess of 100 calories a day – the amount one finds in 10 jellybeans, one cup of soda, or a quarter of a muffin – might translate into over 10 lbs weight gain in a single year^e^[59,60]. Moreover, individuals systematically underestimate their caloric intake [61-63], as well as the number of decisions made about food each day. Of the estimated 220 daily decisions about food, individuals typically recall making only 20 [64]. As Novak and Brownell argue, the overwhelming majority of such “mindless” dietary choices are vulnerable to the influence of the food environment [1,64]. Overconsumption of calories therefore is simply a logical response to obesogenic environments that promote caloric overconsumption through cost [65,66] portion sizes [59,67], accessibility [68-70], the eating environment [71], information deficiencies [72], or even misleading or incorrect information [73]. Given the reality that the food choices of many individuals are constrained by a multitude of factors [58,74], it is difficult to accept the argument that most obese individuals rationally choose to overconsume, and thus are personally responsible for their obesity. By any measure, individuals have little to no control over their food environments [46,75], environments scholars have characterized as toxic [1].

Confronting the toxic food environment should be the focus of public health departments, practitioners, policymakers, and researchers concerned with rising rates of obesity. Starting at the municipal level, ample can be done to change the food environment; New York City is an obvious example of such change [76]. To increase public support for policies to limit the problem of excessive energy input, the frame caloric overconsumption must be accompanied by a concurrent understanding of food environments both as a risk to health and a risk beyond any individual’s control. Of course, the frame caloric overconsumption will not entirely eliminate the idea of personal responsibility in food choices. Instead, what it does is allow for a more critical discussion about how meaningful an individual’s food choices are. While an individual is able to make some choices (e.g., brand preference), the caloric content, portion size, convenience, cost, availability, and marketing of products, among many other variables, are all beyond their control. Reframing obesity as caloric overconsumption is the first step, making the case for why something needs to be done about the food environment.

### The promise and challenges of reframing

The uptake of policies and interventions aimed at preventing obesity has been slow, as policymakers are left with the difficult task of sifting through competing evidence, assessing claims from a variety of stakeholders, and gauging public opinion. The frame obesity only serves to complicate matters. Because it permits the inclusion of policies that are aimed at energy output (e.g., tax incentives to encourage physical activity, modifying built environments to facilitate physical activity), the frame obesity simply adds more noise to the policy cacophony and presents an opportunity for industry to distort or exaggerate the role of energy expenditure. This is where the promise of reframing obesity as caloric overconsumption lies.

Lawrence argues that policy environments shift and become more conducive to accepting policy solutions when an issue is reframed as a systemic problem, particularly when risks are shown to be involuntary [19]. This was the case in tobacco control [28]. Tobacco companies historically have adopted a rhetorical framework that emphasizes personal responsibility for smoking to deflect any hint of culpability for tobacco-related harms. When new evidence emerged about the harms of second-hand smoke, however, the health consequences of tobacco use were no longer considered to be solely the result of individual choices. This resulted in a shift in framing dynamics in favour of tobacco control. For a similar shift to occur in obesity prevention it is not necessary to generate any new evidence, as there is ample evidence demonstrating the involuntary risks created by the food environment. Instead, what is required is for the frame caloric overconsumption, and its emphasis on food environments, to become dominant. This will help to minimize some of the framing competition by excluding energy output and notions of “balance”^f^, as well as other “causes” of obesity that have spurious or limited impact, such as genetics [1], that shift perceptions of risk.

There are several challenges facing the adoption of the frame caloric overconsumption. For one, the reframing strategy used in tobacco is not entirely translatable. Obesity prevention has a more complicated aim than tobacco control, which was concerned with a single harmful behaviour, tobacco use. Moreover, obesity has no obvious second-hand effects that immediately affect the health of others [19], is indirectly linked to numerous eating behaviours [3], many of which are not intrinsically harmful, and, unlike tobacco use, people do need to eat [9]. Nevertheless, scholars have begun to identify some foods as “toxic” [77] or “pathogenic” [78], and the involuntariness of food consumption might even be characterized as “second-hand eating” in some instances. A second challenge is that reframing might have some unintended negative impacts, at least initially. The issue of obesity presently has considerable currency, politically and publicly, and it is possible that reframing could impede existing momentum. Similarly, the media might find the issue of caloric overconsumption less appealing than its sexier counterpart, obesity. That said, obesity will still remain the problem (outcome) needing to be addressed, the frame caloric overconsumption is simply a better way to understand why the problem exists and for identifying what preventive measures should be taken.

The greatest challenge, however, will come from the food and beverage industry. Even if policymakers are willing to address caloric overconsumption, they will continue to face the power of food and beverage industry [46]. At present, the industry has been opposed to most, if not all, food policies that would bring about meaningful changes in obesity rates [79]. It has also invested considerable resources to control the frame [2]. Any reframing that places more responsibility on industry is likely to be met with considerable opposition. Industry can also be expected to emphasize the personal responsibility on the part of those that do overconsume. Controlling the frame will not require the industry to disprove that energy input’s role in obesity; the industry will simply have to create doubt. As frames do not require evidence, industry can dominate framing competitions by manufacturing uncertainty [80], a tactic perfected by tobacco companies. This is why generating more evidence to support obesity prevention policies is ultimately insufficient. Evidence requires interpretation and application, opening the door to further framing competitions, where the salient issue is often not scientific method or empirical facts but becomes about the credibility of claimants [81] or a struggle over morality [3]. It would seem, in this regard, that the industry’s efforts are being rewarded. Obesity is still largely regarded as an individual-level problem, with obese individuals stigmatized as lazy (too little energy output) or undisciplined (too much energy input) [82]. The enactment of “common-sense consumption legislation” (aka Cheeseburger bills), preventing claimants from litigating against food companies for causing obesity [83], suggests the food and beverage industry’s frames are also dominating politically and legally.

Notwithstanding these challenges, reframing holds considerable potential to shift the policy environment, particularly if public health advocates strategically present the frame of caloric overconsumption as a logical and foreseeable outcome of toxic food environments. Importantly, reframing will allow for policymakers to more readily decipher the current policy cacophony by identifying policies that will address energy input. Consider some of the more popular policies and interventions that are presently aimed at obesity prevention but would specifically address caloric overconsumption: tax schemes on “unhealthy” foods or ingredients, subsidies on fruits and vegetables, stricter labelling requirements, zoning by-laws to prevent high caloric foods being available near schools, banning marketing to children, and outright bans on some ingredients (e.g., trans fat). Aimed at caloric overconsumption rather than obesity, these policies promise to be more effective. Effectiveness would not be measured by reductions in obesity rates, which may take years to develop (and is a point on which many current obesity prevention policies fail), but by more proximal (and temporally closer) outcomes, such as reductions in calories consumed or diet quality. Moreover, if caloric overconsumption is understood as an involuntary risk, these policies could be framed as necessary to create a caloric environment that empowers individuals to make positive choices and avoid overconsumption. As such, these policies are likely to be more politically palatable and garner more public support [84] as they would promote autonomy and choice rather than infringe civil liberties, a complaint often levied against obesity prevention policies [9,46].

## Summary

This paper contends that the noise of the obesity policy cacophony can be reduced, and that policymakers will be able to make better sense of the remaining policy options, if the problem of obesity is reframed. Caloric overconsumption, particularly as a logical response to toxic food environments, is presented as a more appropriate frame for the discussion than obesity. With so many possible policies and interventions to scrutinize, rather than add to the noise that already exists it is advisable to focus on the most promising route to effecting change. Policies that aim to reduce the consumption of calories may not be sufficient on their own, but this paper contends they hold the greatest promise for reducing the rates of obesity. Additionally, environmental factors related to food are presented as the most promising route for such change. While the problem of obesity continues to loom large, and the challenges that need to be overcome are formidable, reframing obesity as caloric overconsumption is the type of small change that can have a significant impact on public opinion and policy.

## Endnotes

^a^ Obesity is itself a cause of other diseases, including some types of cancer, type 2 diabetes, and heart disease. ^b^ Barry and colleagues [32] have examined how obesity metaphors, such as ‘epidemic’, affect support for public policy, arguing that the use of metaphors can influence support for obesity policies. The use of metaphors in framing is not considered here. ^c^ Consider PepsiCo’s classification of its products as falling into three board categories: “good-for-you”, “better-for-you”, and “fun-for-you” [1]. ^d^ A common strategy in obesity prevention is to focus on children as they have the least control over their food environments. While a noteworthy approach, this may reinforce notions of personal responsibility for dietary behaviours in adults. After all, the implication is that children need protection from toxic food environments because they lack the necessary faculties to make rational decisions about consumption. ^e^ If a single pound of fat is 3500 calories, an excess of 100 calories a day for a year would equal 36,500 calories, or roughly 10 lbs. Although there is good reason to be wary of 3500 kcal per pound rule, particularly with respect to weight loss [41], it is nevertheless a useful benchmark for considering weight gain. ^f^ It is important to stress that the value and importance of physical activity is not in question here. As noted above, physical activity is important for many aspects of physical and mental health.

## Competing interests

The author has no competing interests.

## Author’s information

JS is a Doctor of Juridical Science candidate at the Faculty of Law, University of Toronto, a Canadian Institutes of Health Research Vanier Canada Graduate Scholar, and a Canadian Institutes of Health Research Health Law, Ethics and Policy Fellow. His dissertation is examining the use of private law as a tool in obesity prevention, specifically tort litigation for caloric overconsumption.

## Pre-publication history

The pre-publication history for this paper can be accessed here:

http://www.biomedcentral.com/1471-2458/12/1042/prepub
